# Effects of unilateral vs. bilateral flywheel-based complex training on change-of-direction and sprint performance in elite male volleyball players

**DOI:** 10.3389/fphys.2026.1786449

**Published:** 2026-04-14

**Authors:** Jiaoqin Wang, Jiajun Lan, Zhikai Qin, Chen Liang

**Affiliations:** 1 School of Physical Education and Coaching Science, Capital University of Physical Education and Sports, Beijing, China; 2 Fujian Normal University, School of Physical Education and Sport Science, Provincial University Key Laboratory of Sport and Health Science, Fuzhou, China; 3 Department of Physical Education, Peking University, Beijing, China

**Keywords:** resistance training, plyometric exercise, agility, athletic performance, volleyball, eccentric overload

## Abstract

**Background:**

Flywheel training has been shown to enhance lower-limb power; however, evidence comparing unilateral and bilateral complex training, especially among elite volleyball players, remains limited.

**Methods:**

Twenty-four male college volleyball players were randomly divided into three groups: unilateral flywheel complex training (UFT, n=8), bilateral flywheel complex training (BFT, n=8), or a control group (CON, n=8) that continued their regular technical training only. The intervention lasted 8 weeks, with 2 sessions each week. Performance measures included linear sprint times (5 m, 10 m, 30 m), change-of-direction skills (T-test, 5-0-5, volleyball-specific agility), and movement endurance (seven T-tests and repeated 30 m runs). A two-way repeated-measures ANOVA was performed to identify differences between groups and across time points (pre-test vs. post-test).

**Results:**

Significant group × time interactions were observed across sprint, COD, and repeated-movement tests (p < 0.05). UFT demonstrated greater improvements than both BFT and CON in short-distance sprint (5–10 m), COD performance, and repeated-movement measures. BFT also improved performance relative to CON in selected outcomes, particularly the 30 m sprint and repeated tests, although improvements were generally smaller than those observed in UFT.

**Conclusion:**

Eight weeks of unilateral flywheel-based complex training resulted in the greatest improvements in short sprints, COD, and movement endurance compared to bilateral training and the control group. Although bilateral training also enhanced performance, the gains were comparatively smaller. These findings support the effectiveness of UFT as a strategy for improving short-distance acceleration and multidirectional movement performance relevant to volleyball match play.

## Introduction

Lower-limb movement in volleyball mainly involves quick, multidirectional actions within approximately 0.7 to 2 meters (Hank et al., 2019). These movements require explosive starts, a low ready stance, and smooth transitions between initiation, locomotion, and deceleration phases (Gadre et al., 2019). Hank et al. (2019) further highlight that elite players most often perform forward, lateral, and diagonal steps, emphasizing the importance of efficient directional changes to meet offensive and defensive needs. Consistent with these findings, such short-range movements are quite common in matches, accounting for approximately 91.8% (Hank et al., 2019). Beyond movement patterns, the physical demands of competition can also be measured by total distance covered, which varies with match duration—from 1,221 ± 327 m in 3-set matches to 1,757 ± 462 m in 4-set matches, with the difference being statistically significant. On average, players cover about 400–450 meters per set, with only minor but non-significant differences between sets. Unlike other team sports where distance is heavily influenced by direct ball contact, volleyball players mainly depend on anticipatory movements in response to ball trajectory and the actions of teammates or opponents. Within this framework, setters, outside hitters, and middle blockers often perform many short-distance movements, which are crucial for tactical execution and quick directional changes (Mroczek et al., 2014). Therefore, targeted training to improve short-range lower-limb movement ability could be essential for maximizing tactical efficiency and overall match performance.

Furthermore, evidence shows that larger displacement distances, especially laterally, greatly decrease reception effectiveness, emphasizing the need to optimize initial positioning and train receivers to reduce unnecessary movement (MacKenzie et al., 2012; Paulo et al., 2016). Short-range lower-limb movement ability relies on reaction speed, initiation and braking skills, explosive power, and coordination (Sheppard and Young, 2006; Davids et al., 2013; Nimphius et al., 2016). These factors highlight that short-range movement efficiency can be limited by individual traits, stressing the need for targeted training to improve performance.

Regarding training interventions, traditional methods provide certain benefits but also face notable limitations. For example, Multi-Directional Speed Training (MDST) enhances COD, acceleration, and deceleration in youth athletes (Young and Rogers, 2014; Born et al., 2016); However, its impact on short-distance sprint performance in adult athletes appears to be modest, with more significant improvements typically seen through strength and power training (Doma et al., 2020; Cin et al., 2021). Resistance training establishes this crucial foundation, but bilateral training modes, while permitting higher external loads (Gabriel et al., 2006), are limited by the bilateral deficit (BLD) (Hay et al., 2006). Unilateral resistance training, by better mimicking volleyball’s single-leg actions, has shown greater improvements in COD and sprinting performance (Santana, 2001; Fisher and Wallin, 2014).

Flywheel (iso-inertial) training delivers eccentric overload, effectively compensating for the low eccentric stimulus common in traditional resistance training methods (Maroto-Izquierdo et al., 2017a; Beato et al., 2021b). This adaptive resistance has been connected to improvements in sprint speed and braking ability (Berg and Tesch, 1994; Fiorilli et al., 2020; Stojanović et al., 2021). Furthermore, plyometric training (PT) enhances these benefits by strengthening the stretch–shortening cycle and increasing explosive power (Behm et al., 2017). When resistance and plyometric exercises are combined, complex training (CT) uses post-activation potentiation (PAP) to improve strength and neuromuscular adaptations (Aagaard, 2003). Evidence indicates that combined training (CT) is more effective than single-mode approaches for improving strength, power, and mobility (MacDonald et al., 2012). Notably, emerging research suggests that unilateral CT may lead to greater improvements in agility than bilateral CT (Escobar Hincapié et al., 2021). Overall, these findings imply that combining flywheel training with CT—especially in a unilateral format—may enhance volleyball-specific movement performance. However, direct comparisons between unilateral and bilateral flywheel-based complex training in elite volleyball players remain scarce. Moreover, it remains unclear whether unilateral training provides superior benefits specifically for short-distance acceleration and change-of-direction tasks that are highly relevant to volleyball match demands.

Therefore, the present study aimed to compare the effects of unilateral and bilateral flywheel-based complex training on: (1) short-distance acceleration and change-of-direction (COD) performance, which are highly relevant to volleyball-specific movement demands, and (2) longer sprint capacity and repeated sprint performance, which reflect general lower-limb athletic ability. By distinguishing between sport-specific and general performance outcomes, this study sought to better clarify the transfer potential of unilateral versus bilateral flywheel-based training in trained volleyball players.

## Methods

### Experimental approach to the problem

**Figure 1:**
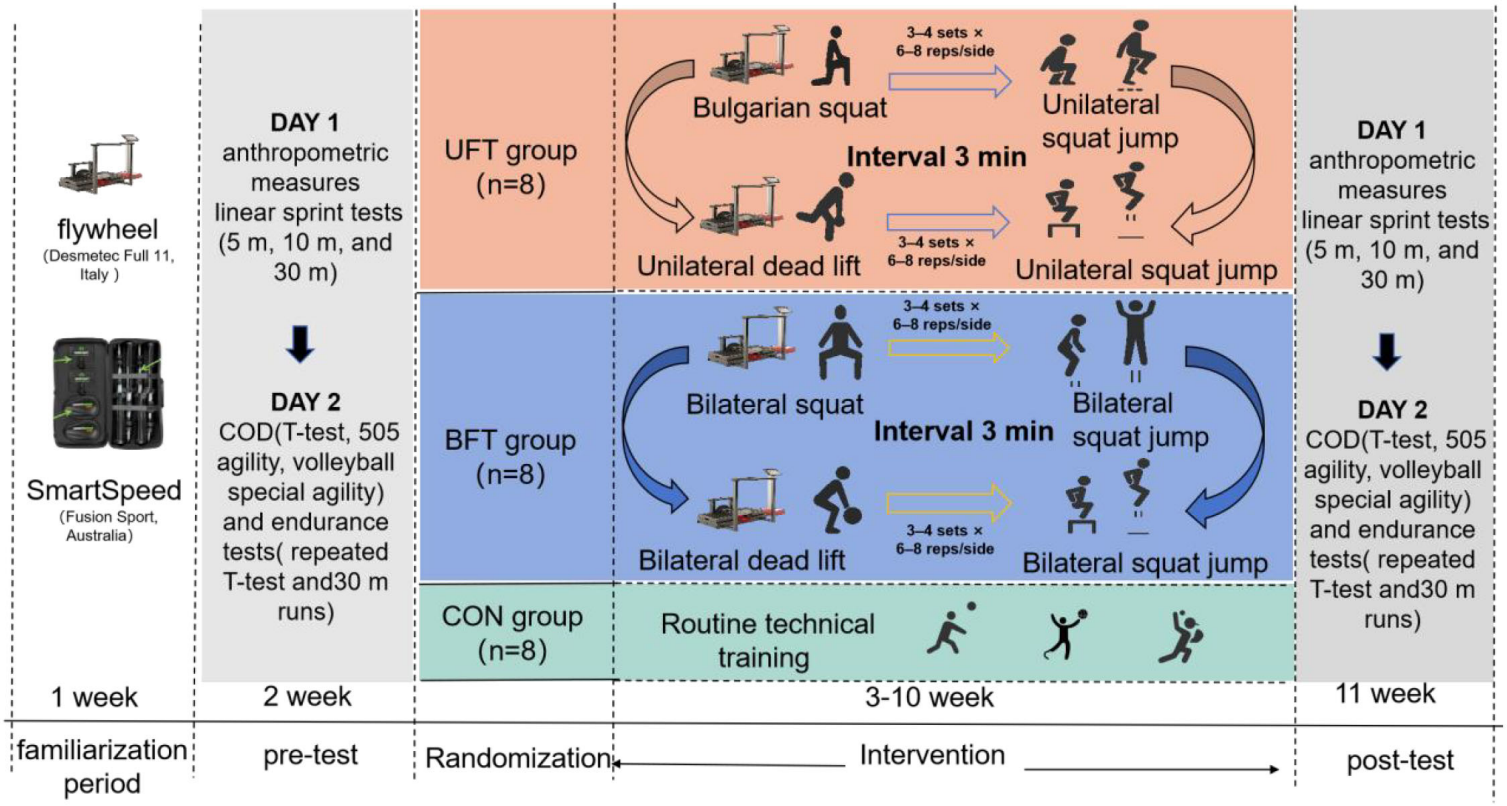
Overall study design, including all measurement points.

The current study aimed to evaluate the effects of unilateral and bilateral flywheel-based complex training on sprinting, COD, and movement endurance performance in elite male volleyball players (**Figure 1**). Subjects were randomly assigned to three groups: unilateral flywheel complex training (UFT, n = 8), bilateral flywheel complex training (BFT, n = 8), or a control group (CON, n = 8) that only continued with regular technical training. Testing was carried out before and after the 8-week intervention across two separate sessions. On Day 1, anthropometric measurements and linear sprint tests (5 m, 10 m, and 30 m) were performed. On Day 2, COD and endurance tests were conducted, including the T-test, 505 agility, volleyball-specific agility, repeated T-test, and repeated 30 m sprints.

All participants completed a one-week familiarization period before baseline testing to ensure proper technique and adaptation to the flywheel device. During the intervention, the UFT and BFT groups trained twice a week under the supervision of certified strength and conditioning professionals. Each session included 3 to 4 sets of 6 to 8 reps of the assigned exercises, with controlled rest periods (1 to 2 minutes between sets and 3 minutes between different exercise types). The UFT group performed unilateral Bulgarian split squats and unilateral Romanian deadlifts on a flywheel device, followed by unilateral half-squat jumps and drop jumps, while the BFT group completed the bilateral versions. The CON group continued with only volleyball technical training.

All training sessions were consistently conducted in the afternoon to minimize circadian variation. Participants were instructed to maintain their usual diet and lifestyle while avoiding additional resistance training or physical therapy during the study period. All testing and training sessions were supervised by the same team of investigators to ensure proper technique and safety compliance.

### Subjects

An *a priori* power analysis was performed using G*Power 3.1.9.2 (F tests, repeated measures ANOVA, within–between interaction). The parameters were set at α=0.05, 1−β=0.80, three groups, two measurements, a correlation among repeated measures of 0.50, and ϵ=1. The expected effect size was set at f=0.35 (η²_p_≈0.11), indicating a moderate-to-large group×time interaction. This estimate was derived from effect sizes reported by Núñez et al. (2018) (Núñez et al., 2018), who examined the effects of unilateral versus bilateral eccentric-overload training on power and COD in team sports players. Under these assumptions, the total sample size needed was N = 24, which matched the number of participants recruited for the present study and yielded an actual power of 0.82.

All participants were registered as Category A (High-level) student-athletes and competed in the China University Volleyball League Competition, the highest tier of collegiate competition in China. Notably, all participants held official National Level I or Elite Athlete (Jianjiang) certifications, representing the top echelon of athletic proficiency in the country. Participants had an average of over 4–5 years of systematic, professional-grade training experience. Given their elite competitive standing and verified technical mastery, they were operationally defined as elite university-level athletes.

During the training period, no subjects withdrew from either the control group or the training groups for unrelated personal reasons, resulting in a final sample of 24 subjects for data analysis. **Table 1** displays the characteristics of the subjects at baseline, and no significant differences were found among groups at the start (p > 0.05).

**Table 1:** Descriptive characteristics of the study sample.

Variable	BFT (n=8)	UFT (n=8)	CON (n=8)	F	P
Age (years)	19.63 ± 1.188	19.88 ± 0.835	20.13 ± 1.553	0.332	0.721
Height (cm)	188.84 ± 6.446	190.38 ± 5.878	192.63 ± 6.886	0.705	0.505
Weight (kg)	78.5 ± 14.976	79 ± 9.695	82.63 ± 9.303	0.300	0.744
Training experience (years)	6.5 ± 1.512	7.38 ± 1.685	6.88 ± 2.642	0.382	0.687

Inclusion criteria were as follows: (i) holding at least a National Level I athlete certification (including Elite Athlete/Jianjiang grade); (ii) having a minimum of 4–5 years of systematic volleyball training experience; (iii) absence of musculoskeletal injury or illness within the six months prior to the study; (iv) participation in at least two volleyball-specific training sessions per week; (v) completion of a minimum of 4–6 weeks of structured resistance training experience (Arazi et al., 2014); and (vi) the ability to perform a back squat equal to 1.5 times body mass and complete five back squats at 60% of body mass within 5 seconds (Pérez-Castilla et al., 2022). This study received ethical approval from the Capital University of Physical Education and Sports, with examination and approval number 2023A075. Prior to starting the experiment, all participants provided written informed consent. All risks associated with the procedures were explained before testing began, and the study was conducted following the guidelines outlined in the Declaration of Helsinki (1964).

### Testing procedures

Participants were familiarized with the equipment, training, and testing procedures three days before data collection. Pre- and post-tests were conducted one week before and after the eight-week training period. On the testing day, each participant completed a 20-minute warm-up, which included 10 minutes of jogging followed by 10 minutes of stretching and ballistic movements; then, they performed the specific warm-up required for each test (Arazi et al., 2014).

### Anthropometric measures

Height and body mass were measured using a standard stadiometer and a calibrated scale, with participants dressed in standardized volleyball competition attire and shoes. Height was recorded to the nearest 0.1 cm, and body mass was recorded to the nearest 0.1 kg.

### Performance measures

Performance measures were categorized into two domains: (1) volleyball-relevant acceleration and change-of-direction (COD) performance (5 m sprint, 10 m sprint, T-test, 505 agility test, and volleyball-specific agility (VSA) test); and (2) general sprint capacity (30 m sprint and repeated 30 m sprint), which reflect broader lower-limb speed and sprint endurance characteristics.

Short-distance acceleration (5–10 m) and COD tests were considered highly relevant to volleyball-specific movement demands, whereas longer sprint distances (30 m) and repeated sprint tests were interpreted as indicators of general sprint capacity.

All electronically timed tests were conducted using SmartSpeed timing gates (Fusion Sport Pty Ltd, Australia). For manually timed assessments, the same experienced evaluator conducted all trials to minimize inter-rater variability.

Unless otherwise specified, three trials were performed for each test, and the best performance was retained for analysis. For repeated sprint and repeated agility protocols, the mean time across repetitions was use.

### Linear sprint tests

#### 30-m sprint test

**Figure 2:**
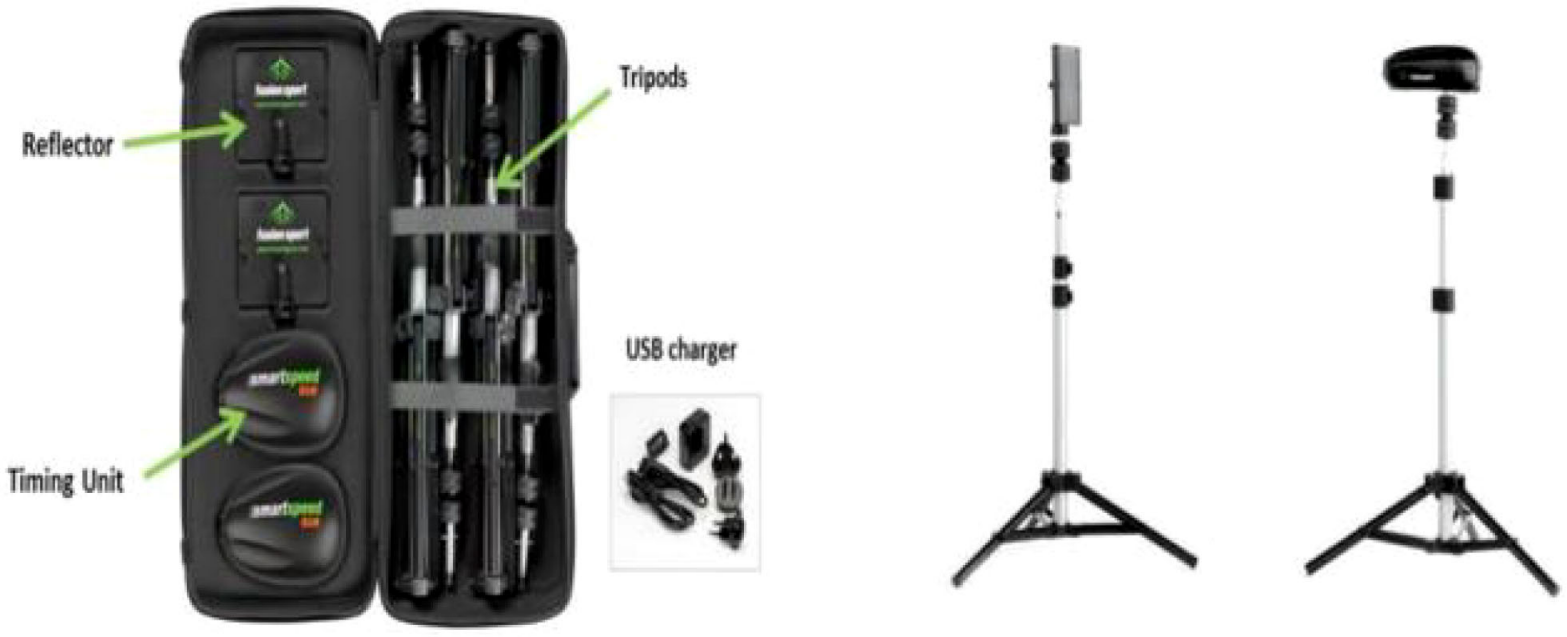
Smartspeed.

The 30-m sprint test was performed using portable infrared timing gates (SmartSpeed Pro; Fusion Sport, Australia; **Figure 2**), positioned at 0 m, 5 m, 10 m, and 30 m. To prevent premature activation of the first gate, participants began 30 cm behind the start line. Timing commenced when the infrared beam at the start line was broken and stopped when the athlete crossed the 30-m gate.

Each athlete completed three trials with 2–3 min rest between attempts. The fastest trial was retained for analysis.

#### Repeated 30-m sprint test

The repeated sprint test followed the same setup as the single 30-m sprint. Athletes performed five consecutive 30-m sprints with standardized recovery intervals. The mean time across the five repetitions was used for subsequent analysis.

### Change-of-direction performance tests

#### T-test

**Figure 3:**
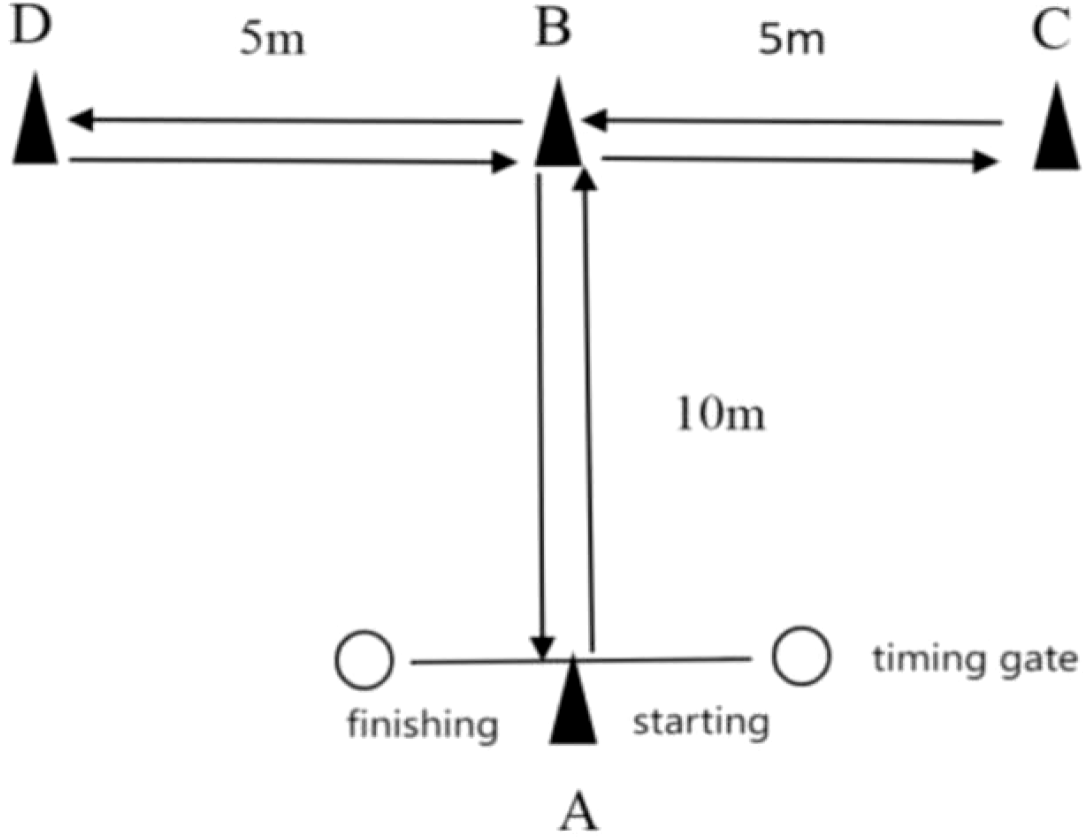
Schematic representation of T-test.

The T-test was conducted using a standardized version as described in the existing literature (Miller et al., 2006). Distances were converted from yards to meters, resulting in a 10 × 10 m testing area. Participants faced forward throughout the test. The testing route used in this study was based on the work of Miller et al. (**Figure 3**) (Miller et al., 2006). Timing gates were positioned 0.75 m above the surface and 3 m apart at the starting line. Timing commenced when the participant passed through the gates and ended upon returning through the finish line.

Three trials were performed with 3–5 min rest between attempts. The best performance was recorded for analysis.

#### Repeated T-test

The repeated T-test was conducted using the same procedure. Participants completed seven consecutive repetitions. The mean time across the seven trials was used for analysis.

#### 505 agility test

**Figure 4:**
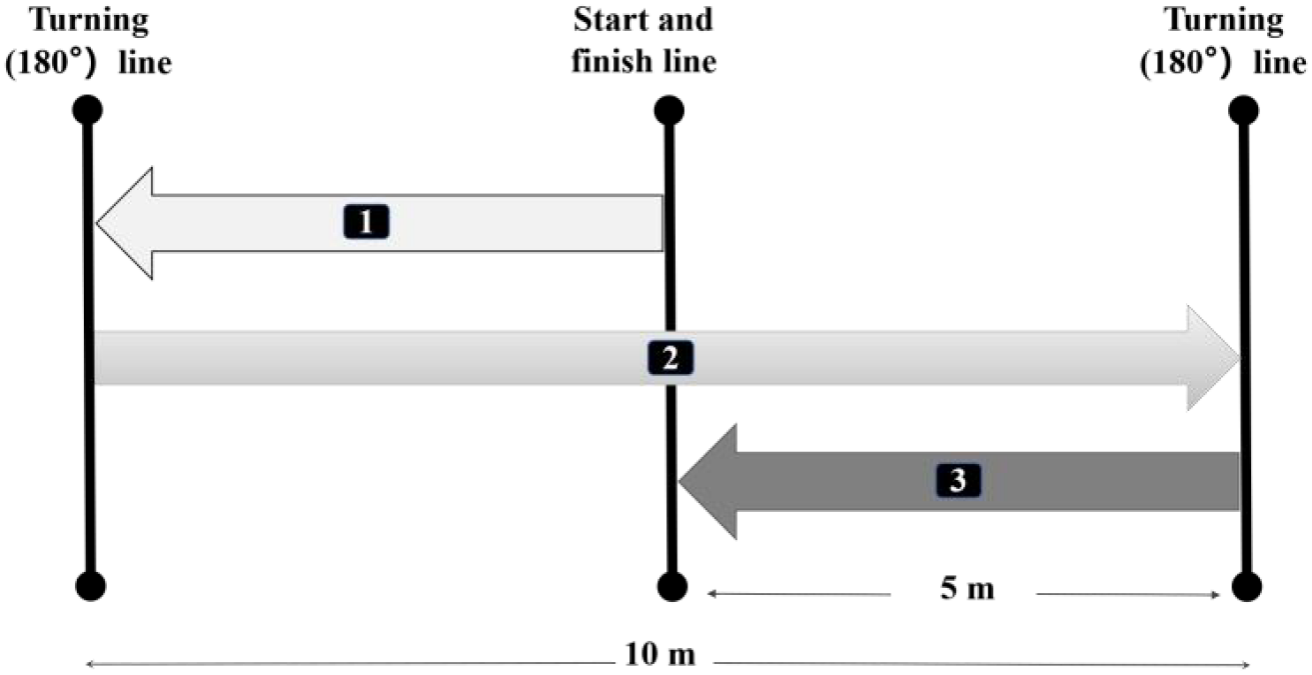
Schematic representation of 505 agility test.

The 505 agility test was conducted over a straight 15-meter distance, with two electronic timing gates positioned at the 10-meter mark. Participants sprinted 15 meters in a straight line and executed a 180° turn using either their left or right leg, with three trials for each leg. Timing began when the participant broke the gate at 10 meters and ended when they crossed the gate again after completing the turn at 15 meters. Three trials were performed for each leg, with 2–3 min rest between trials. The fastest trial was selected for analysis (**Figure 4**).

#### VSA test

**Figure 5:**
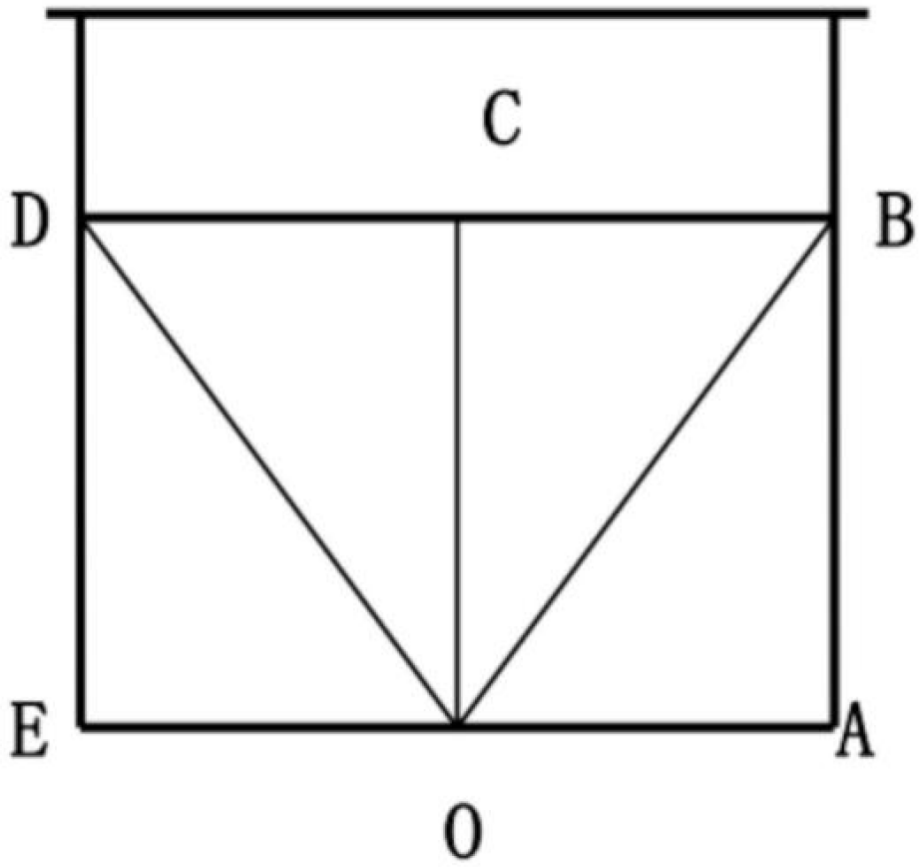
Schematic representation of VSA test.

The VSA test was conducted on the volleyball court, with the starting point ‘O’ at the middle of the end line. Points C, B, and D were positioned at the intersections of the midpoint of the attack line with the attack line and both sidelines, forming an isosceles right triangle (**Figure 5**). A weighted bottle was placed at each endpoint. The athlete started timing by pressing down the bottle at point O using a stopwatch (Tianfu PC894, Shenzhen, China). The test was conducted sequentially along the OA-OB-OC-OD-OE path, with the final timing recorded when the bottle at point O was pressed down. If any bottle was not activated, the trial was repeated. Three trials were performed, and the fastest time was retained for analysis. All manually timed VSA trials were conducted by the same evaluator.

### Flywheel resistance exercise training

Before the intervention, participants completed two familiarization sessions that included fitting the harness, explaining safety precautions, and practicing the designated exercises to ensure technical proficiency and reduce potential learning effects.

The flywheel-based complex training lasted 8 weeks, with two sessions per week (16 sessions total) conducted on a flywheel device (Desmotec Full 11, Italy). Sessions were scheduled at least 48 hours apart to optimize recovery–adaptation balance, particularly given the concurrent technical volleyball training. All training was performed in a fitness laboratory under the supervision of an experienced researcher and a certified strength and conditioning coach. Sessions were consistently held in the afternoon (14:30–15:30) to minimize circadian variability.

Each session began with a standardized 15-minute warm-up consisting of light jogging and submaximal dynamic contractions.

### Training structure

In the present study, the intervention is defined as flywheel-based complex training, in which resistance exercises performed on a flywheel device (characterized by eccentric overload) were paired with biomechanically similar plyometric movements. Thus, the term “flywheel training” refers to the resistance modality, “eccentric overload” describes the mechanical characteristic of the flywheel device, and “complex training” denotes the structured pairing of resistance and plyometric exercises.

Unilateral Flywheel Training (UFT) Participants performed:

3–4 sets of flywheel Bulgarian split squats (6–8 repetitions), immediately followed by unilateral squat jumps (6–8 repetitions);

3–4 sets of flywheel unilateral Romanian deadlifts (6–8 repetitions), followed by unilateral drop jumps (6–8 repetitions).

Bilateral Flywheel Training (BFT)Participants performed:

3–4 sets of flywheel squats (6–8 repetitions), followed by bilateral squat jumps (6–8 repetitions);

3–4 sets of flywheel bilateral Romanian deadlifts (6–8 repetitions), followed by bilateral drop jumps (6–8 repetitions).

During all flywheel exercises, participants were instructed to contract explosively during the concentric (ascent) phase and to resist the strap as much as possible during the eccentric (descent) phase, when the strap rewound and caused eccentric overload. Strong verbal encouragement was provided throughout the exercises. Participants were reminded to use only light resistance during the first third of the eccentric phase and to gradually increase to maximum braking force afterward to optimize eccentric overload (Beato et al., 2019).

All exercises were performed in this sequence under strict supervision.

A 3-minute inter-set rest interval was implemented between complex pairs. This duration was selected to allow partial phosphagen system recovery while managing neuromuscular fatigue and preserving potentiation effects, in accordance with recovery–fatigue management principles.

### Loading prescription and individualization

The training intervention was structured to optimize neuromuscular adaptations while ensuring systematic fatigue management and adherence to the principle of progressive overload.

1. Training Frequency and Recovery.

The choice of two sessions per week (separated by 48–72 hours) was implemented to provide an adequate recovery window. This frequency aligns with established strength and conditioning principles for athletic populations, allowing for optimal supercompensation and high-quality mechanical output in each session while minimizing the risk of overtraining or cumulative neural fatigue.

2. Loading Prescription and Individualization.

To ensure individualized and precise loading, we accounted for the velocity-dependent nature of flywheel resistance, which contrasts with traditional constant-external-load training. Based on findings by Spudić et al. (R² = 0.96) (Spudić et al., 2020), barbell movement velocity at 80% 1RM was measured using a GymAware Power Tool during half-squats, Romanian deadlifts, and Bulgarian split squats. These values were used to calibrate the corresponding inertial load on the flywheel device.

3. Repetition Scheme and Volume.

The 6–8 repetition range per set (3–4 sets per exercise) was selected to target strength–power development. According to NSCA-CSCS guidelines, ~80% 1RM typically corresponds to this repetition bracket. This scheme strategically balances high force production demands with the preservation of movement velocity—a critical factor for enhancing sprint acceleration and change-of-direction performance. The plyometric component mirrored this volume (3–4 sets of 6–8 repetitions) to maintain biomechanical similarity and consistent training density within each complex pairing.

4. Progressive Overload and Fatigue Management.

To maintain a continuous mechanical stimulus as participants adapted to eccentric loading, flywheel inertia was increased by 0.025 kg·m²every two weeks. Furthermore, training volume was progressively increased from six repetitions per set (Weeks 1–4) to eight repetitions (Weeks 5–8). This incremental progression was designed to enhance the physiological stimulus and prevent performance plateaus without inducing excessive cumulative fatigue. To ensure consistent eccentric loading, participants performed three preliminary repetitions before each set to accelerate the flywheel to the target velocity; these were not included in the prescribed repetition count.

### Monitoring and compliance

To monitor internal training load, participants rated their perceived exertion using the CR10 Rating of Perceived Exertion (RPE) scale immediately after each set. The average score across sets was recorded as the weekly RPE value. Participants were instructed to avoid strenuous physical activity and abstain from caffeine or alcohol for at least 12 hours before each session. Meanwhile, the control group continued their usual volleyball technical training throughout the intervention period and did not perform additional strength or plyometric training. The control group participated in two regular team training sessions per week (approximately 90–120 minutes each), primarily consisting of technical drills (passing, setting, spiking, serving), tactical exercises, and instructional match play. No structured strength or plyometric training was included during the intervention period.

### Statistical analysis

Analyses were conducted using SPSS v.23.0 (SPSS Inc., IBM, China). The Shapiro–Wilk, Levene, and Mauchly tests were used to assess the data for normality, homogeneity, and sphericity, respectively. For pre-test between-group comparisons, a univariate analysis of variance (ANOVA) was used. The effects of the experimental intervention were assessed using a two-way repeated-measures ANOVA, with group (UFT, BFT, and CON) and time (pre-test versus post-test at 8 weeks) as factors. In cases where a statistically significant interaction or main effect was found, *post hoc* comparisons were conducted using the Bonferroni correction to identify mean differences. Statistical significance was established at P ≤.05. Mean ± SD values are reported in the text. Percentage change was calculated as (Pre − Post)/Pre × 100. The effect size (ES) of the training was calculated using partial eta squared, as recommended by Cohen (1965) (Cohen, 1965). Cohen classified ES values as small (0.01), medium (0.06), and large (0.14) to represent modest, moderate, and substantial effects, respectively. No data points were excluded from the analyses, and no formal outlier removal procedures were applied. All analyses were conducted using the complete dataset of 24 participants.

## Results

To facilitate magnitude-based interpretation and improve reporting transparency, **Table 2** summarizes pre–post mean values, absolute changes, percentage changes, and corresponding 95% confidence intervals for all performance variables. These data complement the statistical interaction effects presented above and allow a clearer evaluation of the practical significance of training adaptations.

**Table 2:** Pre–post changes and magnitude of performance adaptations.

Variable	Group	Pre (Mean ± SD)	Post (Mean ± SD)	Δ (Pre - Post)	%Δ	95% CI of Δ
T-test (s)	BFT	10.785 ± 0.514	10.211 ± 0.531	0.574	5.32	[0.138, 1.009]
	UFT	10.384 ± 0.407	9.411 ± 0.369	0.973	9.37	[0.681, 1.264]
	CON	10.727 ± 0.593	10.856 ± 0.522	-0.129	-1.20	[-0.455, 0.197]
VSA Test (s)	BFT	15.901 ± 0.548	15.274 ± 0.512	0.628	3.95	[0.057, 1.198]
	UFT	15.459 ± 0.726	14.218 ± 0.516	1.241	8.03	[0.777, 1.705]
	CON	15.862 ± 0.896	16.425 ± 1.117	-0.563	-3.55	[-1.266, 0.141]
505 Agility (s)	BFT	2.574 ± 0.152	2.423 ± 0.170	0.151	5.88	[0.074, 0.229]
	UFT	2.473 ± 0.142	2.220 ± 0.145	0.253	10.21	[0.143, 0.362]
	CON	2.566 ± 0.070	2.612 ± 0.136	-0.046	-1.80	[-0.172, 0.079]
5 m Sprint (s)	BFT	1.018 ± 0.092	0.958 ± 0.129	0.060	5.90	[-0.037, 0.157]
	UFT	1.018 ± 0.102	0.768 ± 0.022	0.250	24.57	[0.177, 0.323]
	CON	1.058 ± 0.122	1.098 ± 0.161	-0.040	-3.78	[-0.182, 0.102]
10 m Sprint (s)	BFT	2.164 ± 0.263	2.019 ± 0.215	0.145	6.70	[0.004, 0.286]
	UFT	2.248 ± 0.221	1.620 ± 0.064	0.628	27.92	[0.470, 0.785]
	CON	2.136 ± 0.358	2.208 ± 0.263	-0.071	-3.34	[-0.384, 0.241]
30 m Sprint (s)	BFT	4.519 ± 0.298	4.178 ± 0.182	0.341	7.55	[0.147, 0.535]
	UFT	4.349 ± 0.209	3.815 ± 0.281	0.534	12.27	[0.224, 0.844]
	CON	4.586 ± 0.252	4.536 ± 0.229	0.050	1.09	[-0.049, 0.149]
Repeated T-test (s)	BFT	12.044 ± 0.592	11.144 ± 0.650	0.900	7.47	[0.394, 1.406]
	UFT	11.726 ± 0.714	10.289 ± 0.559	1.437	12.26	[0.892, 1.983]
	CON	11.965 ± 0.739	12.106 ± 0.595	-0.141	-1.18	[-0.448, 0.165]
Repeated 30 m Sprint (s)	BFT	4.774 ± 0.277	4.630 ± 0.328	0.144	3.01	[0.040, 0.247]
	UFT	5.095 ± 0.247	4.232 ± 0.075	0.862	16.93	[0.673, 1.052]
	CON	4.976 ± 0.298	4.975 ± 0.262	0.001	0.03	[-0.062, 0.065]

### COD performance

**Figure 6:**
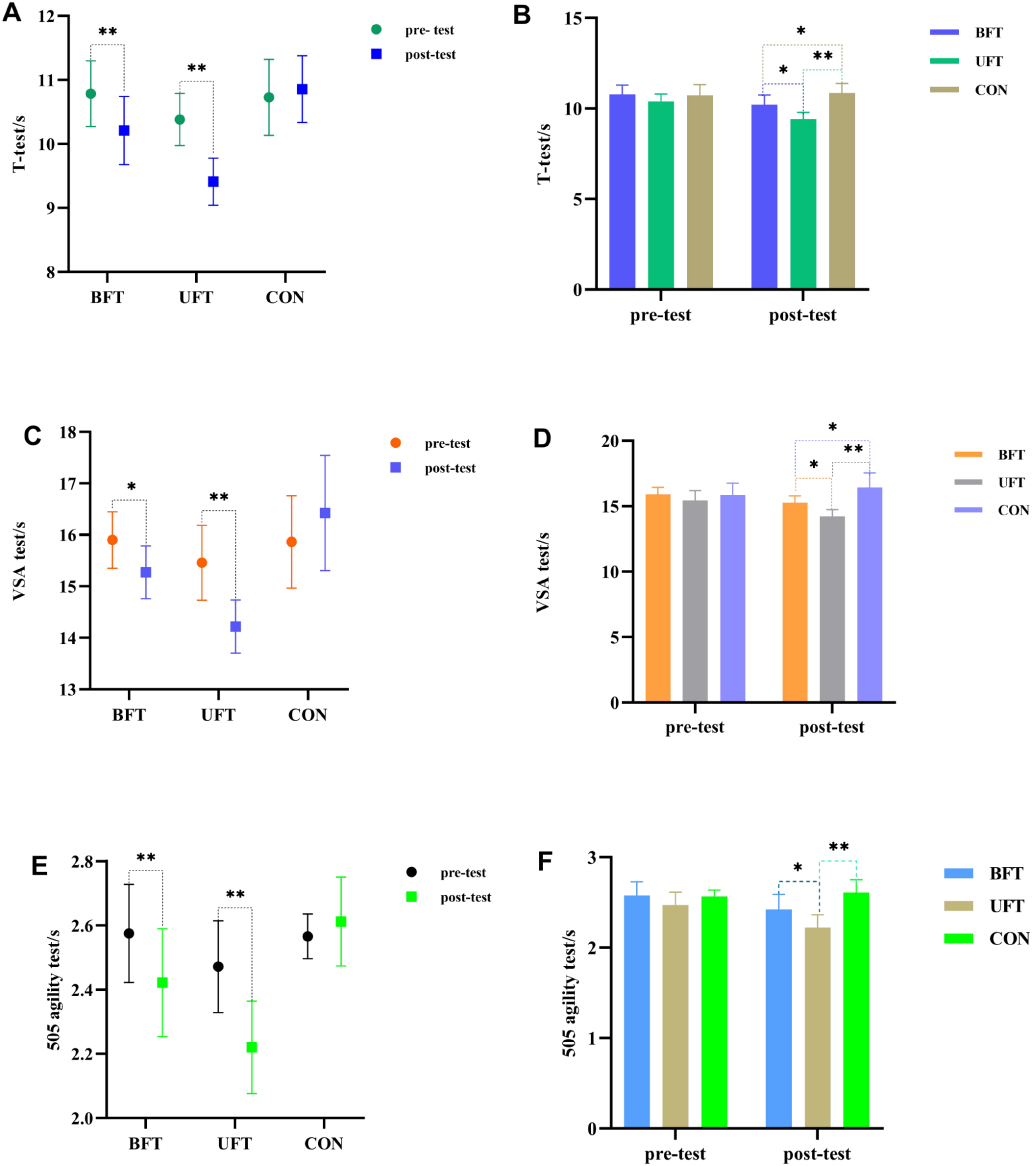
Within- and between-group results of change-of-direction (COD) tests. UFT, unilateral flywheel complex training; BFT, bilateral flywheel complex training; CON, control group (technical training only). **p < 0.01. *p < 0.05, indicate significant differences between groups at post-test. **(A)** Pre- and post-test T-test times by group. **(B)** Between-group T-test comparisons at post-test. **(C)** Pre- and post-test VSA test times by group. **(D)** Between-group VSA test comparisons at post-test. **(E)** Pre- and post-test 505 agility test times by group. **(F)** Between-group 505 agility test comparisons at post-test.

The two-way repeated ANOVA showed that all three COD tests (T-test, VSA test, and 5-0–5 agility) exhibited significant main effects of time, group, and time × group interaction (all p < 0.01, η² > 0.30), indicating that training effects differed between groups and that the intervention appeared to be effective (**Figures 6A–F**). Detailed absolute changes (Δ), percentage changes (%Δ), and 95% confidence intervals are reported in **Table 2**.

Regarding the T-test (**Figures 6A, B**), the largest improvement was observed in the UFT group (η² = 0.665), followed by the BFT group (η² = 0.408), while the CON group showed no notable changes. Post-test comparisons further revealed that UFT was significantly faster than both BFT and CON (p < 0.05), with BFT also surpassing CON (p < 0.05). Specifically, UFT recorded the fastest post-test time (9.41 ± 0.37 s), compared to BFT (10.21 ± 0.53 s) and CON (10.86 ± 0.52 s).

In the VSA test (**Figures 6C, D**), UFT showed the greatest improvement (η² = 0.543), BFT showed moderate improvement (η² = 0.233), and CON showed minimal change (η² = 0.196). At post-test, both UFT and BFT were significantly faster than CON (p < 0.05), with UFT also performing significantly better than BFT (14.22 ± 0.52 s vs. 15.27 ± 0.51 s, p < 0.05).

Finally, the 505 agility test (**Figures 6E, F**) showed that UFT achieved the greatest gains (η² = 0.594), BFT had smaller improvements (η² = 0.354), and CON showed no significant change (η² = 0.047). Post-test comparisons confirmed that UFT was significantly faster than both BFT (2.22 ± 0.14 s vs. 2.42 ± 0.17 s, p < 0.05) and CON (2.61 ± 0.14 s, p < 0.001), while the difference between BFT and CON approached significance (p = 0.058).

### Linear sprint performance

**Figure 7:**
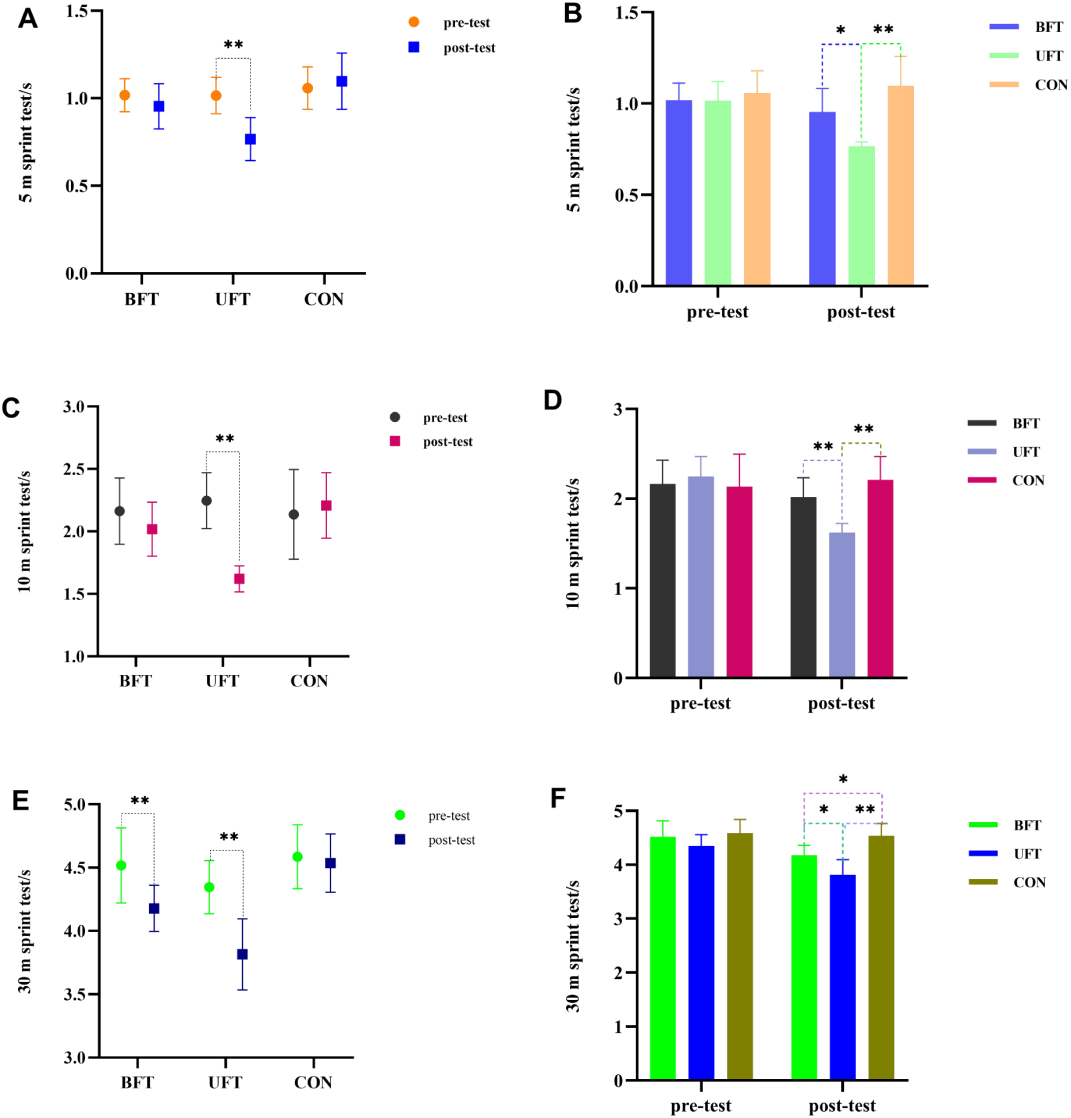
Within- and between-group results of linear sprint performance tests. UFT, unilateral flywheel complex training; BFT, bilateral flywheel complex training; CON, control group (technical training only). **p < 0.01. *p < 0.05, indicate significant differences between groups at post-test. **(A)** Pre- and post-test 5 m sprint times by group. **(B)** Between-group 5 m sprint comparisons at post-test. **(C)** Pre- and post-test 10 m sprint times by group. **(D)** Between-group 10 m sprint comparisons at post-test. **(E)** Pre- and post-test 30 m sprint times by group. **(F)** Between-group 30 m sprint comparisons at post-test.

The two-way repeated ANOVA analysis revealed significant main effects of time, group, and a group × time interaction for all three sprint tests (5 m, 10 m, and 30 m) (all p < 0.01, η² > 0.36), indicating that sprint performance changes differed across groups and that the intervention appeared to be effective(**Figures 7A–F**). Corresponding magnitude-based data are summarized in **Table 2**.

For the 5 m sprint (**Figures 7A, B**), *post-hoc* comparisons revealed that UFT showed the largest improvement, outperforming both CON (0.77 ± 0.12 s vs. 1.10 ± 0.16 s, p < 0.001) and BFT (0.77 ± 0.12 s vs. 0.95 ± 0.13 s, p = 0.015). There was no significant difference between BFT and CON (p = 0.078).

In the 10 m sprint (**Figures 7C, D**), UFT again demonstrated the largest improvement, running significantly faster than both BFT (1.62 ± 0.10 s vs. 2.02 ± 0.22 s, p < 0.001) and CON (2.21 ± 0.26 s, p < 0.001), while BFT and CON showed no significant difference (p = 0.212).

Regarding the 30 m sprint (**Figures 7E, F**), UFT showed the greatest improvements, followed by BFT, while CON showed no significant change. At post-test, UFT was significantly faster than both BFT (3.82 ± 0.12 s vs. 4.18 ± 0.18 s, p < 0.05) and CON (4.54 ± 0.12 s, p < 0.01), and BFT was also faster than CON (4.18 ± 0.18 s vs. 4.54 ± 0.12 s, p = 0.018).

### Movement endurance performance

**Figure 8:**
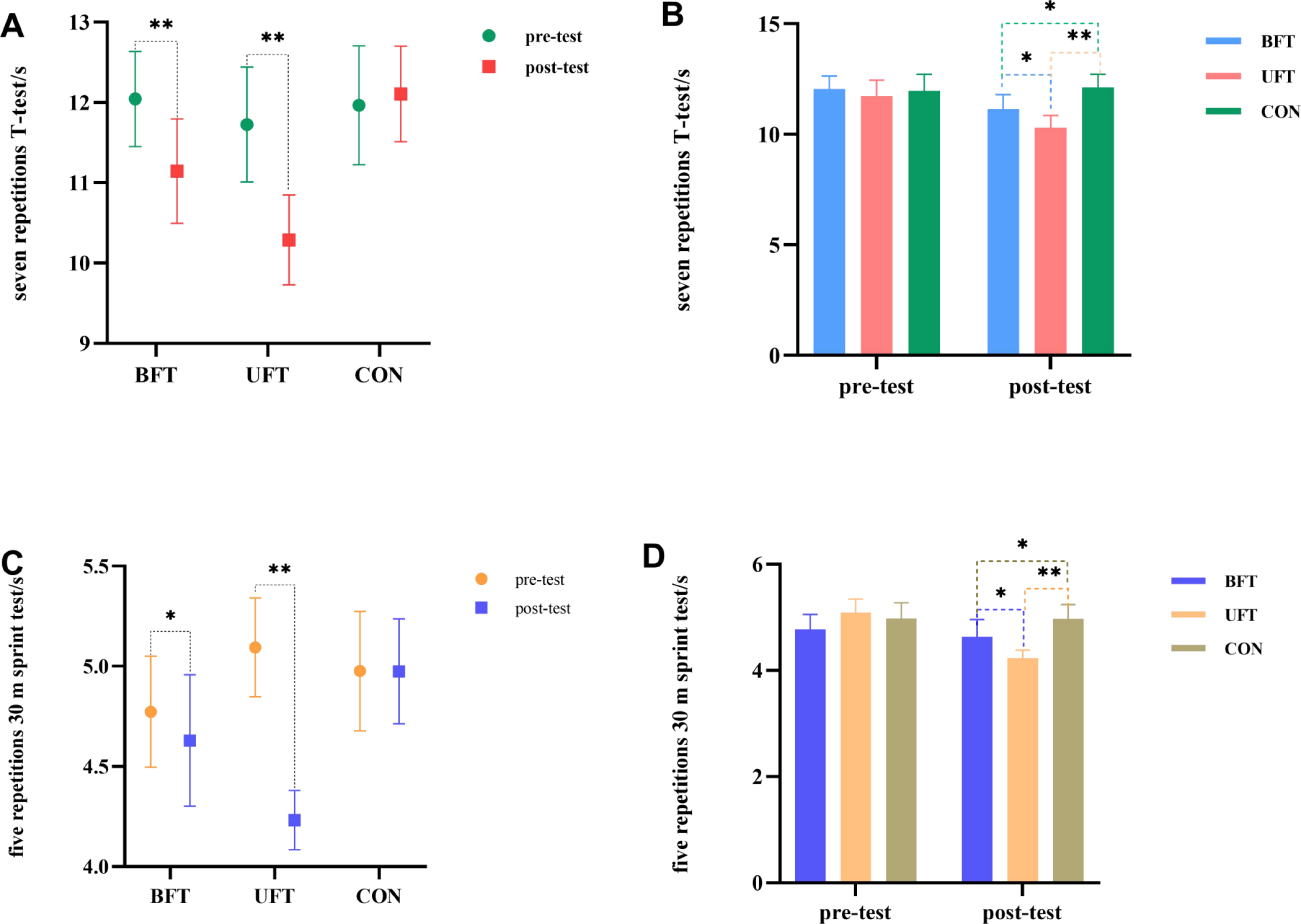
Within- and between-group results of movement endurance tests (repeated T-test and repeated 30 m sprint). UFT, unilateral flywheel complex training; BFT, bilateral flywheel complex training; CON, control group (technical training only). **p < 0.01. *p < 0.05, indicate significant differences between groups at post-test. **(A)** Pre- and post-test repeated T-test times by group. **(B)** Between-group repeated T-test comparisons at post-test. **(C)** Pre- and post-test repeated 30 m sprint times by group. **(D)** Between-group repeated 30 m sprint comparisons at post-test.

The two-way repeated ANOVA showed significant group × time interactions for both the repeated T-test and the repeated 30 m sprint (all p < 0.05, η² > 0.28), indicating different training effects across groups (**Figures 8A–D**). For magnitude-based interpretation of these changes, see **Table 2**.

In the repeated T-test (**Figures 8A, B**), UFT showed the largest improvements (η² = 0.718), and performed significantly better than both CON (10.29 ± 0.56 s vs. 12.11 ± 0.60 s, p < 0.001) and BFT (10.29 ± 0.56 s vs. 11.14 ± 0.65 s, p < 0.05). Additionally, BFT outperformed CON (11.14 ± 0.65 s vs. 12.11 ± 0.60 s, p < 0.05).

In the repeated 30 m sprint (**Figures 8C, D**), UFT again demonstrated the largest gains (η² = 0.946), followed by BFT (η² = 0.280), while no significant changes were observed in CON (η² = 0.000). At post-test, UFT was significantly faster than both CON (4.23 ± 0.15 s vs. 4.98 ± 0.26 s, p < 0.001) and BFT (4.23 ± 0.15 s vs. 4.63 ± 0.33 s, p < 0.05). Additionally, BFT also outperformed CON (4.63 ± 0.33 s vs. 4.98 ± 0.26 s, p < 0.05).

## Discussion

The present study compared the effects of unilateral and bilateral flywheel-based complex training on sprint and change-of-direction performance in elite male volleyball players. The primary findings were that unilateral training was associated with greater improvements in short-distance acceleration (5–10 m) and change-of-direction performance compared with bilateral training and technical training alone. Both unilateral and bilateral interventions improved 30 m sprint and repeated sprint performance; however, the magnitude of change was consistently greater in the unilateral group. In contrast, the control group exhibited minimal or trivial changes. The slight negative changes observed in the control group likely reflect the absence of a targeted neuromuscular stimulus and the inherent variability of short-duration sprint performance, rather than systematic performance deterioration. Collectively, these findings indicate that unilateral flywheel-based complex training may provide a more effective neuromuscular stimulus for enhancing rapid acceleration and multidirectional performance, which directly addresses the primary aim of comparing unilateral and bilateral training adaptations in volleyball players.

These results should be interpreted within the framework of the principle of specificity. Volleyball match play is primarily characterized by short accelerations (often <5 m), rapid lateral adjustments, and reactive multidirectional movements rather than sustained maximal sprinting over longer distances. Therefore, improvements observed in 5–10 m sprint and COD tests likely demonstrate stronger ecological relevance to volleyball performance, whereas enhancements in 30 m and repeated 30 m sprint performance should be interpreted as improvements in general sprint capacity rather than strictly sport-specific movement ability. This distinction strengthens the theoretical coherence of the present findings and supports the study’s objective of differentiating sport-specific adaptations (short-distance acceleration and COD) from general sprint capacity outcomes.

Two main mechanisms may help explain the present findings. Importantly, it should be noted that the current study did not directly assess neuromuscular or morphological adaptations; therefore, the following interpretations are based on existing literature rather than direct physiological measurements. At the neural level, unilateral resistance training has been shown to induce cross-education effects through cortical and corticospinal adaptations, whereby strength and motor improvements in one limb may transfer partially to the contralateral limb (Carroll et al., 2006; Manca et al., 2017). Previous research also suggests that flywheel resistance training, characterized by continuous concentric–eccentric transitions, may increase neuromuscular activation and coordination demands (Maroto-Izquierdo et al., 2017a). Although neural activity was not measured in the present study, it is plausible that such adaptations contributed to the superior improvements observed in the unilateral group. At the muscular level, the eccentric overload inherent in flywheel training improves braking ability and energy absorption, thereby enhancing control during quick decelerations and directional shifts (Beato et al., 2021a). These abilities are especially important in volleyball, where athletes repeatedly perform quick movements (e.g., blocking, lateral shuffling, and rapid recovery steps) that require eccentric strength and dynamic control stability (Ziv and Lidor, 2010). Unilateral training more effectively mimics the asymmetrical support and rotational control required during these actions, potentially making it particularly relevant for volleyball-specific demands.

These findings are consistent with and extend the current literature. Beato et al. (2019) demonstrated that eight weeks of flywheel training enhanced speed and power in soccer players; however, they did not compare unilateral and bilateral training modes (Beato et al., 2019). Núñez et al. (2018) reported that both unilateral and bilateral eccentric-overload training improved performance in team sport athletes, with unilateral training having greater effects on horizontal jumps and COD, while bilateral training contributed more to hypertrophy and maximal strength (Núñez et al., 2018). Similarly, Belegišanin et al. (2025) compared unilateral and bilateral flywheel training in youth basketball players and found that both methods improved physical performance, but unilateral protocols produced better gains in agility- and acceleration-related tasks, further emphasizing the functional specificity of unilateral training in multidirectional movement sports (Belegišanin et al., 2025). This study supports these patterns by showing that unilateral protocols were associated with greater improvements in agility- and acceleration-related tasks. Additionally, Askling et al. (2003) found that eccentric-dominant training reduces injury risk in soccer players by enhancing hamstring control, suggesting that eccentric overload training may not only improve performance but also provide protective benefits (Askling et al., 2003). Maroto-Izquierdo et al. (2017) further emphasized the multi-dimensional benefits of flywheel training, especially for sports that require frequent high-intensity, explosive movements (Maroto-Izquierdo et al., 2017b). Our findings extend this evidence to volleyball, suggesting sport-specific applicability.

Although several outcomes—particularly in repeated sprint performance—demonstrated large partial eta squared (η²_p_) values, these effect sizes should be interpreted with caution given the relatively small sample size (n = 8 per group). In small-sample experimental designs, magnitude estimates can be susceptible to inflation and are highly sensitive to within-group variability. Furthermore, despite the inclusion of a familiarization period, potential learning effects or repeated testing exposure across sessions cannot be entirely excluded as contributing factors to performance gains. Additionally, inherent measurement variability, especially in manually timed assessments, may have influenced the observed magnitudes. Consequently, we have reported absolute and percentage change values alongside 95% confidence intervals to provide a more transparent and robust contextual interpretation of the training adaptations.

From a practical perspective, these findings provide practical insights for volleyball conditioning. Since the sport emphasizes quick, short-distance sprints, frequent COD, and repeated efforts while fatigued (Gabbett and Georgieff, 2007; Sheppard et al., 2007), coaches may consider prioritizing UFT to enhance transfer to match performance. In practice, this may involve implementing unilateral flywheel–plyometric pairings twice weekly during preparatory phases, using moderate-to-high inertial loads with progressive overload. Additionally, coaches might consider including unilateral flywheel exercises during the preparatory and strength phases, preferably alongside plyometric and COD drills, to develop integrated programs that improve neuromuscular adaptation and movement efficiency. Furthermore, load prescriptions can be tailored based on player positions (e.g., middle blockers who require greater multidirectional mobility), potentially enhancing the precision of conditioning strategies. Therefore, the practical recommendations derived from this study directly stem from the observed superiority of unilateral training in volleyball-relevant performance tasks, further supporting the applied implications of the study’s primary objective.

## Conclusion

Eight weeks of unilateral flywheel-based complex training was associated with greater improvements in short-sprint, change-of-direction, and repeated-movement (endurance) performance compared to BFT or technical training alone in elite male collegiate volleyball players. Although BFT also yielded significant improvements compared to the control group, these gains were less pronounced than those observed with unilateral training. These results suggest that unilateral flywheel-based complex training may be an effective and practical approach to enhance short-distance acceleration and multidirectional movement performance relevant to volleyball match play and may be prioritized in conditioning programs. Coaches may consider including unilateral flywheel exercises along with plyometric and sport-specific change-of-direction drills, while also tailoring inertial load and volume based on player roles to maximize transfer. Future research with larger, mixed-sex samples, longer follow-ups, mechanistic assessments, and injury tracking is warranted to further examine these results and refine training strategies.

### Limitations

Several limitations should be acknowledged. First, the relatively small sample size (n = 8 per group) may constrain the generalizability of the findings and increase the susceptibility to effect size inflation, particularly for large η²_p_ values. Second, although familiarization sessions were conducted, potential learning effects or repeated testing exposure cannot be entirely ruled out as contributing factors to performance improvements. Third, the use of manual timing in certain assessments may introduce inherent measurement variability. Future research employing larger cohorts and automated timing systems is warranted to validate and extend these preliminary results.

## Data Availability

The raw data supporting the conclusions of this article will be made available by the authors, without undue reservation.
